# A randomized, open phase IV exploratory clinical trial to evaluate the efficacy and safety of acupuncture on the outcome of induction of ovulation in women with poor ovarian response: A study protocol for a randomized controlled trial

**DOI:** 10.1097/MD.0000000000011813

**Published:** 2018-08-24

**Authors:** Hoyoung Lee, Tae-Young Choi, Eun Hyoung Shim, Jiae Choi, Jong Kil Joo, Bo Sun Joo, Myeong Soo Lee, Jun-Yong Choi, Ki-Tae Ha, Sooseong You, Kyu Sup Lee

**Affiliations:** a Clinical Medicine Division, Korea Institute of Oriental Medicine, Yuseong-gu, Daejeon; b Integrative Health Promotion Team, Korea Health Promotion Institute, Jung-gu, Seoul; c Department of Obstetrics and Gynecology, Pusan National University, Seo-gu, Busan; d Infertility Institute, Pohang Women's Hospital, Pohang-si, Gyenonsangbuk-do; e Department of Internal Medicine, Korean Medicine Hospital of Pusan National University, Yangsan, Gyeongsangnam; f Department of Korean Medical Science, School of Korean Medicine and Korean Medical Research Center for Healthy Aging, Pusan National University, Yangsan, Gyeongsangnam, Republic of Korea.

**Keywords:** acupuncture, anti-Müllerian hormone, antral follicle count, IVF-ET, oocytes, poor ovarian responder

## Abstract

**Introduction:**

Women with infertility who have a poor ovarian responder (POR), characterized by a low number of retrieved oocytes after ovulation induction, often have a significantly reduced pregnancy rate after in vitro fertilization-embryo transfer (IVF-ET), due to the few transferred embryos. Acupuncture is a form of Korean Traditional Medicine. It involves the insertion of a microscopic needle at a specific point in the body, known as an acupuncture point or an acupoint. In this study, our purpose is to investigate how acupuncture affects the retrieval of mature oocytes after ovulation induction in patients with POR.

**Methods and analysis:**

This study will be a randomized clinical trial comprising an IVF-ET trial and an IVF-ET trial after acupuncture. Seventy patients will by enrolled and randomly assigned to either of the 2 groups. The study subjects will be required to be diagnosed as having POR. Participants will be divided into 2 groups: IVF-ET single treatment group, and acupuncture and IVF-ET combined treatment group. The study subjects will be required to participate in a 15-week trial involving 16 acupuncture treatments over a period of approximately 2 months before ovulation induction for oocyte retrieval. The primary assessment of all participants will be comparing the number of oocytes.

**Result:**

This treatment will be a therapeutic model for POR.

**Discussion:**

Our results will provide patients with POR as well as complementary and alternative medicine professionals, such as Korean medicine doctors, about the potential role of acupuncture in the treatment of POR. This will improve the quality of life in women with infertility and provide an important treatment option for patients with POR. Further studies can be performed to determine the optimal treatment for POR.

## 1 Introduction

As World Health Organization (WHO) defined, infertility is a disease of the reproductive system defined by the failure to achieve a clinical pregnancy after 12 months or more of regular unprotected sexual intercourse.^[1]^ Also, if a patient does not become pregnant despite having a normal marriage for 1 year, the condition is defined as “infertility.” Infertility is reported to be present in approximately 10% to 20% of the population in the reproductive age group.^[2]^ In recent years, various factors, such as increased marriage age, labor intensity, and pollution, have been pointed out as causes for the high infertility rate; these affect approximately 40% of infertile couples.^[3]^ In approximately 15% of patients, the cause of infertility is disordered ovulation, while in 30% to 40%, it is obstruction of the fallopian tubes and/or issues in the abdominal cavity. There is an increasing demand for assisted reproduction techniques, namely in vitro fertilization and embryo transfer (IVF-ET) and artificial insemination, to address this problem.^[4]^ Specially, infertility patients with a poor ovarian responder (POR), which is characterized by a low number of retrieved oocytes after ovulation induction,^[5]^ have a significantly reduced pregnancy rate with IVF-ET due to the low number of transferred embryos.^[6]^ In this case, the most important goal of assisted reproduction technology is increasing the quality and quantity of oocytes.

Although the number of patients with POR is increasing due to various reasons, there is no standard treatment yet. Currently, many patients are using complementary and alternative medical (CAM) treatment therapies, such as acupuncture, moxibustion, and herbal formulas, to increase the success rate of IVF-ET. Acupuncture is a commonly used adjuvant therapy during these CAM therapies.^[7]^

Acupuncture involves the insertion of a microscopic needle at a specific point in the body, known as an acupuncture point or acupoints, as a form of Korean Traditional Medicine (KTM).^[8]^ Acupuncture has a superior safety profile when conducted by a Korean Medicine Doctor (KMD). Ninety percent of patients experience no side effects, and no large-scale events have occurred.^[9]^ In KTM, traditional therapy, including acupuncture and herbal medicine, has been widely used to treat infertility for many years, and many reports on these treatments exist. In 1999, Stener-Victorin et al revealed that acupuncture could increase the IVF clinical pregnancy rate.^[10]^ Since then, randomized clinical trials (RCTs) of acupuncture for treating various causes of infertility, such as POR,^[11]^ thin endometrium,^[12]^ and polycystic ovarian syndrome,^[13]^ have been ongoing. In this study, our purpose is to investigate how acupuncture could affect the retrieval of mature oocytes after ovulation induction in patients with POR.

## 2 Patients and methods

### 2.1 Research evidence and hypothesis

Clinical studies on the effects of acupuncture on the number of retrieved mature oocytes after ovulation induction and pregnancy success rate in patients with POR, especially in China, are underway, and a number of papers, including RCTs, have been published.^[14–16]^ Based on several previous studies, it was hypothesized that acupuncture treatment could increase the number of mature oocytes. To prove this, the current study was designed to investigate the effect of acupuncture treatment on mature oocytes after ovulation induction in patients with POR.

### 2.2 Study design

**Figure 1:**
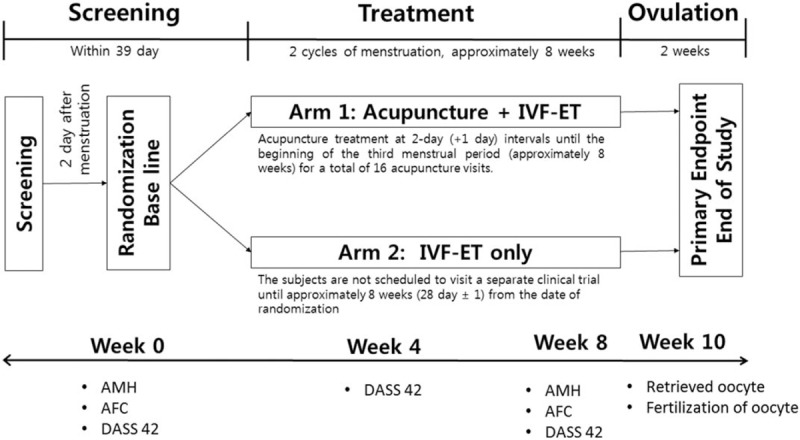
Trial overview.

This study will include only an IVF-ET trial and an IVF-ET trial after acupuncture treatment in the form of RCTs. Seventy patients will by enrolled and randomly assigned into either of the 2 groups of the trial. The trial will be conducted at the infertility center in Pusan National University (PNU, Busan, Republic of Korea, Trial protocol version: KI-17-D-001 Version 2.0, August 8, 2017). The study subjects will be required to be diagnosed as having POR. Participants will be divided into 2 groups: IVF-ET single treatment group, and acupuncture and IVF-ET combined treatment group. The entire flow chart of the study is shown in Fig. 1. The study subjects will be required to participate in a 15-week trial involving 16 acupuncture treatments during a period of approximately 2 months before ovulation induction for oocyte retrieval.

### 2.3 Study population

This study will be conducted in primary care at an infertility center in PNU. Eligible participants will include adult women who are diagnosed with POR and who require induction of ovulation in a procedure-based IVF-ET. Potential participants will be provided with sufficient information about the study, and only those who agree to the study protocol and sign the consent form will be enrolled. The study will be implemented according to the Good Clinical Practice (GCP) terms and the revised version of the Declaration of Helsinki.

### 2.4 Power calculation

This study is a randomized, open phase IV, exploratory clinical trial to assess the efficacy and safety of acupuncture in adult women diagnosed with POR after induction of ovulation. The primary efficacy endpoint will be the number of mature oocytes collected at approximately 10 weeks. The sample size in this study will be calculated by referring to the results of a transcutaneous electrical acupoint stimulation (TEAS) study performed by Zheng et al, because there is no related study, although the same acupuncture study results should be referred to. According to Zheng et al, the number of mature oocytes was counted as 2.38 ± 1.55 in the control group and 4.87 ± 3.47 in the TEAS treatment group. The results of the experimental group were assumed to be the effect of TEAS, and the standard deviation (Stdev) was assumed to be the Stdev of 3.47. Based on the results of this study, 31 subjects were included to secure 80% of power with 5% to the bilateral significance and 1:1 of the dispensing ratio. If the dropout rate is 10%, 35 subjects in each group will be recruited.

### 2.5 Recruitment, randomization

This study is open phase; therefore, random assignment codes will be assigned using the block randomization method. This will be done using SAS (Version 9.3, SAS Institute, Cary, NC), by a statistician who is not directly related to this clinical study. The statistician will distribute the randomization code to the person in charge of the clinical trial institution before commencing the clinical trial.

Subjects who will be enrolled in the study and who will meet the inclusion/exclusion criteria will be assigned to each application group in a randomized sequential envelope according to the random assignment number. This randomization number will be used as the study participant identification code during the trial. The entire process of the study will be directed by the authorized clinical research organization (CRO), CROcent Corporation, Seoul, Korea.

### 2.6 Early termination or dropout

The criteria for early termination or dropout are as follows: withdrawal of consent of research subject or research subject agent; cases in which it is difficult to continue due to an adverse event; case violation of selection criteria or exclusion criteria during the clinical trial; when a combination of drugs is required that affects the safety/efficacy assessment; cases in which the subject cannot be followed up; cases where the trial was judged to be difficult to continue; and cases where the patient falls naturally pregnant

### 2.7 Clinical trial procedure

#### 2.7.1 Screening period

Screening numbers will be assigned to the study subjects who have provided written consent, and then eligibility will be assessed using inclusion/exclusion criteria. The participants will measure the factors: demographic, medical history, and previous drugs. The subjects will be selected from the group based on physical examination, vital signs, physical examination, endometrial examination, endocrine test, laboratory test, serum test, pregnancy test, chest radiographs, and inclusion/exclusion criteria will be assessed to gather information.

#### 2.7.2 Baseline

(1) The baseline is the second day of menstruation. On the second day, information will be collected by performing history confirmation, previous drugs, vital signs, anti-Müllerian hormone (AMH) test, antral follicle count (AFC) using ovarian ultrasound, random assignment, and evaluation with the depression anxiety stress scales (DASS) 42.
(2) One-time after-screening randomization is performed at a ratio of 1:1 eligible subjects on the second day of menstruation.
  - Arm 1: IVF-ET after acupuncture.
  - Arm 2: IVF-ET.

#### 2.7.3 Treatment period

(1) Arm 1: IVF-ET after acupuncture
  The KMD begins acupuncture treatment from the random allocation date of the subjects. The subject will be administered acupuncture treatment at 2-day (+1 day) intervals until the beginning of the third menstrual period (approximately 8 weeks) for a total of 16 acupuncture visits.
(2) Arm 2: IVF-ET
  The subjects are not scheduled to visit a separate clinical trial until approximately 8 weeks (28 day ± 1) from the date of randomization, when they present for oocyte collection.

#### 2.7.4 Ovulation

Arm 1 and Arm 2 groups start 3 cycles of menstruation from the random allocation day, and at the beginning of the third cycle, they visit the institute conducting clinical trials to induce ovulation.

### 2.8 Criteria

#### 2.8.1 Inclusion criteria

The subjects should meet all the following criteria.

1. Sex: female
2. Age: 20 to 45 years
3. Women who have voluntarily signed a written consent to participate in the trial
4. Women who have had infertility for more than 1 year or who have been diagnosed with infertility in a medical institution
5. Women who have been diagnosed with POR (including at least 2 of the following criteria)
  a. Advanced maternal age (≥40 years) or any other risk factor for POR
  b. A previous POR (≤3 oocytes with a conventional stimulation protocol)
  c. An abnormal ovarian reserve test (AMH < 0.5–1.1 ng/mL or total AFC < 5–7 follicles)
6. Women who have the ability and willingness to take the test during the clinical trial period

#### 2.8.2 Exclusion criteria

Patients who meet any of the following conditions cannot participate in the trial:

1. Women who cannot undergo acupuncture treatment for 2 months
2. Women who are taking or administer medicines and procedures related to pregnancy, such as hormones, herbal medicine, and moxibustion treatment
3. Women whose menstrual cycle is irregular (<21 days, more than 40 days)
4. Women whose partner is azoospermia
5. Women diagnosed with ovulation disorders (polycystic ovary syndrome, thyroid disease, hyperprolactinemia, etc.)
6. Women with physical or mental impairment who may be exposed to risk by participating in this clinical trial, who may cause confusion in results, or fail to comply with the requirements of the clinical trial
7. Women who have a neurological or psychologically pathologic history or are currently suffering from a disease
8. Women who cannot be treated with acupuncture due to skin diseases, such as ulcers or eczema
9. Women who have participated in other clinical trials within the past 3 months
10. Women who have a history of drug abuse and alcohol abuse within 5 years prior to clinical trial participation
11. Women who have been diagnosed with infertility due to a deformity of the reproductive organs or leiomyoma
12. Patients for whom the researcher considers that it would be difficult to perform the clinical trial

### 2.9 Intervention

#### 2.9.1 Arm 1: IVF-ET after acupuncture

The interventional protocol and the intensive choice of acupoints were based on the experiences of the KMD and from previous clinical studies (Table 1). See Fig. 1 for a flowchart of trial procedures. The acupoints of CV4 and CV3 will be applied on one side. The acupoints of EX-CA1, SP6, KI3, SP10, ST36, and LR3 will be applied on both sides. All acupoints will be limited to the locations defined by the WHO. During the treatment, participants will lie in the bed, and the KMD will use 75% alcohol pads to sterilize the skin around the acupoints. Subsequently, stainless steel sterilized needles (diameter 0.25 mm, length 3 cm, Dongbang Medical, Korea) will be inserted into the selected acupoints at depths of 0 to 20 mm for 30 minutes. During the acupuncture treatment period, the lower abdomen is irradiated with infrared rays. This group will undergo a total of 16 acupuncture treatments for approximately 8 weeks at 2-day intervals (+1 day) before inducing ovulation.

**Table 1:**
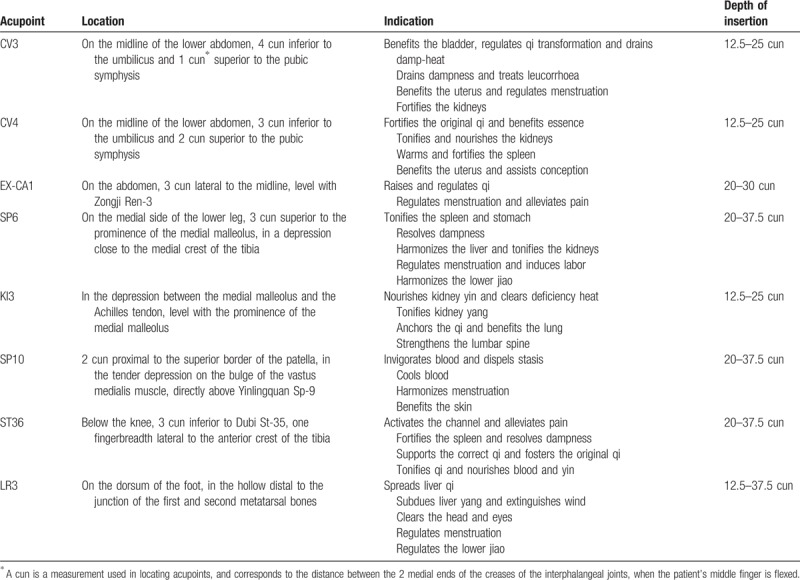
Acupoints used in the acupuncture + in vitro fertilization and embryo transfer group.

#### 2.9.2 Arm 2: IVF-ET

These participants will have no treatment for 2 months to compare with the Arm 1 group. The ovulation induction will then proceed according to the IVF-ET procedure.

### 2.10 Outcome measurements

#### 2.10.1 Primary outcome

The primary outcome will be measured based on the retrieval of mature oocytes at 10 weeks from baseline.

#### 2.10.2 Secondary outcome

At 8 weeks from the baseline of the treatment period, patients will be assessed for AFC and AMH. The DASS-42 score will be measured at 4 and 8 weeks from baseline.

#### 2.10.3 Other outcomes

At 10 weeks from baseline, the in vitro fertilization rate of retrieved oocytes will be assessed.

### 2.11 Assessment analysis

#### 2.11.1 Quality control and data monitoring

Independent of sponsors and competitive interests, CROcent will perform evaluation and monitoring to ensure the results and quality of the clinical trial. During the trial, CROcent will monitor regularly whether the study is proceeding in accordance with the protocol, through the use of all related master files such as documentation, case report forms, informed consent forms, and adverse event reports. When monitoring this trial, we will ensure that the clinical trial will be performed in accordance with the approved protocol and the GCP and related regulation, ensure that case report will be complete and clear, and check with the register.

#### 2.11.2 Data collection, access, and management

The data of this trial will be managed according to the standard work instructions of Pusan University Hospital. Other contents not specified in the trial protocol shall follow the standards of the International Conference on Harmonization of Technical Requirements for Registration of Pharmaceuticals for Human Use (ICH)-Guideline for GCP and the Korean Good Clinical Practice guidelines.

The source document is recorded immediately when the case data are collected. After the source document input is completed, it will be recorded in the case record form. All the documents will be kept safe, so that it can be verified by the relevant government agencies and institutional review board (IRB). Only research colleagues and others who have the approval of the principal investigator have access to all data obtained from this trial.

#### 2.11.3 General analysis

The statistical analysis will be conducted by blinded professional statisticians. Unless otherwise specified, all data will be based on a 2-sided test at a significance level of 5%. The continuous variable gives the number of observation, the mean ± stdev and the median (minimum values, maximum values), while the categorical variable gives the frequency and percentage. If missing values are found in continuous validation variables, they will be analyzed using the last observation carried forward method. At this time, the results before applying the clinical trial device (control randomized) will not be replaced by the results after applying the clinical trial device. If a categorical validation variable has a missing value, it will be considered a failure and included in the analysis. Safety assessment variables will not replace missing values.

#### 2.11.4 Population and clinical history data

The descriptive statistics for each applied group are presented, and the continuous variables will be analyzed with 2-sided *t* test or Wilcoxon rank-sum test according to the satisfaction of normality assumption. Categorical variables will be analyzed to determine whether the cells with an expected frequency of <5 exceed 20% followed by Pearson chi-squared test or Fisher exact test.

### 2.12 Assessment

#### 2.12.1 Primary assessment

The primary endpoint will be the number of mature oocytes harvested at 10 weeks from baseline. The descriptive for each applied group will be presented and analyzed with the 2-sample *t* test or Wilcoxon rank-sum test depending on the satisfaction of normality.

#### 2.12.2 Secondary assessment

1. AFC and AMH level at 8 weeks from baseline
  The descriptive for each applied group will be presented and analyzed with the 2-sample *t* test or
2. DASS-42 score at 4 and 8 weeks compared to baseline
  The descriptive for each applied group will be presented and analyzed with the 2-sample *t* test or Wilcoxon rank-sum test depending on the satisfaction of normality.

#### 2.12.3 Other assessment

(1) The in vitro fertilization rate of retrieved oocytes at 10 weeks from baseline

The frequency and percentage of the applied group will be presented and analyzed with Pearson chi-squared test or Fisher exact test.

#### 2.12.4 Safety assessment

According to method of reporting serious anomalies, abnormal cases collected as parliamentarians will be continuously monitored by an in-house safety manager during the study period. The safety manager will ensure that all significant adverse cases (including the name of the case, the occurrence of the adverse case, the symptom, the date of manifestation, the disappearance, severity, and results) by the clinical trials director or clinical trials representative in the internal safety database in real time. If a new anomaly is reported, the relevance of the case to the medical device within 3 working days will be analyzed to assess whether the duration of the trial is safe.

#### 2.12.5 Patient and public involvement

Patients and/or the public were not involved in the study design and research question. The results of the study, however, will be disseminated through community event at the hospital and patient organizations.

## 3 Research ethics and dissemination

### 3.1 Ethics approval

This trial has received the full ethical approval of the Ethics Committee of Pusan University (D-1706-017-056). The results of the study will be disseminated through a peer-reviewed journal via the media, website, and patient organizations.

### 3.2 Consent

Patients recruited through adverting and voluntary participation will be explained the details of the trial. The patient and the investigator (nurse) must have sufficient interrogation time before signing the clinical trial agreement. This will result in a consent form if the patient agrees to participate in the clinical trial.

### 3.3 Confidentiality

Patient information is anonymized and all relevant investigators must keep the results confidential. The director must keep a signed consent form and prepare a list when confirming the patient's identity is required.

### 3.4 Protocol amendments

The director or sponsor of a clinical trial cannot change the protocol without the other's consent. After the clinical trial has begun, it should only be modified in exceptional cases. If change in the content of the protocol are required, all participants must sign and agree in writing. The revised content must be approved by the IRB committee.

### 3.5 Post-trial care

Those who are assigned to Arm 2 will be provided with 3 months of DHEA (dehydroepiandrosterone), which is known to help POR, if pregnancy fails after IVF-ET. Subsequent care and treatment of subjects who have completed clinical trials will be subject to the general care and treatment guidelines and principles. In the case of damage related to clinical trials, they will be covered by the subject compensation plans and the clinical trial insurance policy.

## 4 Discussion

Acupuncture is widely accepted by Koreans, and it is increasingly requested by patients with infertility. To date, it is difficult to assess the effectiveness of acupuncture due to a lack of RCTs on acupuncture for patients with POR. In this clinical trial, the Arm 1 group has been designed as the intervention group to determine the effectiveness of acupuncture. Patients diagnosed with POR will undergo 8 acupoints treatments for 8 weeks. The patients in the Arm 2 group will be asked not to take any alternative treatments except for rest during the 8 weeks. If this part is not implemented, it will have to be abandoned to complete the research. We will evaluate the quality and number of mature oocytes after acupuncture treatment for patients with POR. We will also observe the clinical pregnancy rate after IVF-ET.

This study can potentially identify whether acupuncture is effective as a POR standard therapy under stringent quality control. Our results will enable patients with POR and CAM professionals, such as KMDs, to determine the potential role of acupuncture in the treatment of POR. This has the potential to improve the quality of life in women with infertility as well as provide an important treatment option for patients with POR. Further studies will be required to determine the optimal treatment for POR, including acupuncture.

## Acknowledgments

The authors would like to thank all the trial participants. The authors are grateful for the support for this study: trial coordinating team (CROcent), surgical staff, nurses, and research departments.

## Author contributions

Hoyoung Lee drafted the manuscript. Tae-Young Choi searched the acupoints in Korea and China. Eun Hyoung Shim and Jiae Choi reviewed and revised the manuscript. Bo Sun Joo, Ki-Tae Ha, and Jun-Yong Choi consulted study design such as acupuncture treatment and IVF-ET schedule. Kyu Sup Lee and Jong Kil Joo performed the IVF-ET after evaluating the oocytes. Sooseong You is the study coordinator and designed the study. All authors read and approved the final version of the manuscript.

**Conceptualization:** Hoyoung Lee, Sooseong You, Kyu Sup Lee.

**Data curation:** Eun Hyoung Shim, Jiae Choi, Myeong Soo Lee.

**Funding acquisition:** Sooseong You.

**Investigation:** Jong Kil Joo, Kyu Sup Lee.

**Methodology:** Tae-Young Choi, Jun-Yong Choi, Bo Sun Joo, Ki-Tae Ha, Kyu Sup Lee.

**Project administration:** Sooseong You.

**Supervision:** Sooseong You, Kyu Sup Lee.

**Writing – original draft:** Hoyoung Lee.

**Writing – review and editing:** Sooseong You.
